# Immune landscape of human prostate cancer: immune evasion mechanisms and biomarkers for personalized immunotherapy

**DOI:** 10.1186/s12885-020-07058-y

**Published:** 2020-06-18

**Authors:** Mayassa J. Bou-Dargham, Linlin Sha, Qing-Xiang Amy Sang, Jinfeng Zhang

**Affiliations:** 1 Department of Chemistry and Biochemistry, Florida State University, Tallahassee, Florida, USA; 2 Department of Statistics, Florida State University, Tallahassee, Florida, USA; 3 Institute of Molecular Biophysics, Florida State University, Tallahassee, Florida, USA

**Keywords:** Prostate cancer, Immunotherapy, Biomarkers, Combination therapy, Immune evasion

## Abstract

**Background:**

Despite recent advances in cancer immunotherapy, the efficacy of these therapies for the treatment of human prostate cancer patients is low due to the complex immune evasion mechanisms (IEMs) of prostate cancer and the lack of predictive biomarkers for patient responses.

**Methods:**

To understand the IEMs in prostate cancer and apply such understanding to the design of personalized immunotherapies, we analyzed the RNA-seq data for prostate adenocarcinoma from The Cancer Genome Atlas (TCGA) using a combination of biclustering, differential expression analysis, immune cell typing, and machine learning methods.

**Results:**

The integrative analysis identified eight clusters with different IEM combinations and predictive biomarkers for each immune evasion cluster. Prostate tumors employ different combinations of IEMs. The majority of prostate cancer patients were identified with immunological ignorance (89.8%), upregulated cytotoxic T lymphocyte-associated protein 4 (CTLA4) (58.8%), and upregulated decoy receptor 3 (DcR3) (51.6%). Among patients with immunologic ignorance, 41.4% displayed upregulated DcR3 expression, 43.26% had upregulated CTLA4, and 11.4% had a combination of all three mechanisms. Since upregulated programmed cell death 1 (PD-1) and/or CTLA4 often co-occur with other IEMs, these results provide a plausible explanation for the failure of immune checkpoint inhibitor monotherapy for prostate cancer.

**Conclusion:**

These findings indicate that human prostate cancer specimens are mostly immunologically cold tumors that do not respond well to mono-immunotherapy. With such identified biomarkers, more precise treatment strategies can be developed to improve therapeutic efficacy through a greater understanding of a patient’s immune evasion mechanisms.

## Background

According to the American Cancer Society, prostate cancer is the most common cancer among US men and the second leading cause of death. The 2018 GLOBOCAN project revealed prostate cancer to be the second most common cancer affecting males worldwide after lung cancer, and the most frequently diagnosed cancer in the US [1]. Local tumors are treated with surgery or radiation therapy and metastatic castrate-sensitive prostate cancer is treated by chemical or physical castration [2, 3]. Cancer relapse and treatment failure are common and result in the progression to castrate-resistant prostate cancer. Therefore, there is a need to develop more effective therapies [4].

Immunotherapy that stimulates a patient’s immune system to target cancer is emerging as a next-generation cancer treatment [5]. Immunotherapy in prostate cancer is currently under investigation to boost the anti-tumor immune response by targeting immunosuppressive molecules [4]. The immunotherapies currently approved by the US Food and Drug Administration (FDA) for prostate cancer is Sipuleucel-T (Provenge) for metastatic castrate-resistant prostate cancer (mCRPC) and Keytruda for solid tumors with mismatch repair genes (MMR) and/or exhibit microsatellite instability (MSI). Despite the improved overall survival achieved by Sipuleucel-T, there was no difference in the progression-free survival in the treatment group compared to placebo [6]. Keytruda on the other hand is given to metastatic prostate cancer patients with MMR/MSI tumors, which represent 5–10% of metastatic patients, only if they have progressed on other treatments and have no satisfactory alternative treatment option. The unsatisfactory results of the immune checkpoint inhibitors, anti-CTLA4 and anti-PD-1 monotherapies [7–9], have led pharmaceutical companies to shift their focus to combined and sequential therapy. Recently, targeting both CTLA4 and PD-1 has resulted in a prostate-specific antigen (PSA) response and objective responses in some patients [6, 10–12]. Several immunotherapies are currently in clinical trial including a few immunotherapy combination treatments such as viral vaccines targeting different cancer antigens (PSA, CEA, and MUC1), viral vaccines with anti-CTLA4 and anti-PD-1, Sipuleucel-T with anti-CTLA4, anti-PD-L1 with interleukin-15 (IL-15) superagonist, IDO inhibitor, and viral vaccines, and other combinations with GM-CSF (Additional file 1). However, the lack of patient inclusion criteria based on predictive biomarkers that could help determine who is likely to respond to treatment hinders the sustained progress towards more effective immunotherapies for prostate cancer.

Recognition of the “cancer-immunity cycle” in the anti-tumor immune response has facilitated a more precise identification of immune evasion mechanisms [13]. The anti-tumor immune response starts with the recognition of the surface antigens on cancer cells by antigen-presenting cells (APCs) (i.e., macrophages and dendritic cells). APCs then prime and activate cytotoxic T lymphocytes (CTLs) to kill cancer cells [14]. The dead cancer cells then release more antigens that activate additional APCs and amplify the anti-cancer immune response by recruiting more immune cells. Thus, a successful response depends on feedback and cycle self-amplification. All of the above steps must be activated to kill cancer cells and achieve success in immunotherapy [14].

The low expression of antigen-expressing molecules, as well as low CTL recruitment and activation, may indicate an impairment in antigen processing and presentation [14–16]. In more extreme cases where all the genes of the cancer immunity cycle are not upregulated compared to normal tissue level, this indicates immunologic ignorance, due to the lack of a danger signal. If the genes responsible for antigen processing and presentation are upregulated but not the cytotoxic molecules secreted by CTLs upon activation, then there is a subsequent impairment in immune cell activation that is potentially caused by tolerance and immunosuppression (e.g., CTLA4, PD-1, PD-L1/2, and TGF-β). In addition, immune cell killing of tumors can be weakened by the cancer cell production of decoy molecules against Fas and TRAIL-induced death pathways (i.e., decoy receptor 3 [DcR3] and decoy receptor 4 [DcR4, *aka* TRAILR4]) [17, 18].

To identify the evasion mechanisms in prostate cancer and the predictive biomarkers for the specific evasion mechanism(s) in a patient, we applied a series of computational methods (sequential biclustering, differential expression, immune cell typing, and machine learning) to prostate cancer RNA-seq data obtained from the cancer genome atlas (TCGA) [19]. The analysis termed an immune evasion mechanism analysis (IEMA), clustered the majority of prostate cancer patients into eight groups based on their expression of immune-related genes [13]. Each of the eight clusters has a distinct set of evasion mechanisms that were simultaneously activated in cancer. Ten biomarkers predictive of the cluster membership of a patient were also selected using a decision tree algorithm.

## Methods

### TCGA prostate cancer dataset and immune gene list

We collected a list of 2000 immune genes from previous publications and gene sets from the Molecular Signatures Database (MSigDB) (Additional file 2) [20–22]. We then checked the RNA-Seq expression in prostate cancer in the Cancer Genome Atlas (TCGA) database (https://www.cancer.gov). The datasets included 498 prostate adenocarcinoma (PRAD) samples and 52 matched non-malignant adjacent normal tissue samples. We generated two data matrices: a cancer matrix (2000 × 498) and non-malignant adjacent normal matrix (2000 × 52). The de-identified clinical information for the patients was also gathered from TCGA.

### Sequential biclustering

To separate the patients into different groups based on their similar gene expression, we used the plaid biclustering package in R, *BCPlaid,* and clustered them sequentially to obtain discrete, non-overlapping subsets of patients [23]. The sequential algorithm continues until no more clusters with at least 5% of the total number of samples can be found [13]. Additional file 3 contains the level of expression of the 2000 immune genes in the identified clusters.

### Immune cell analysis

**Fig. 1:**
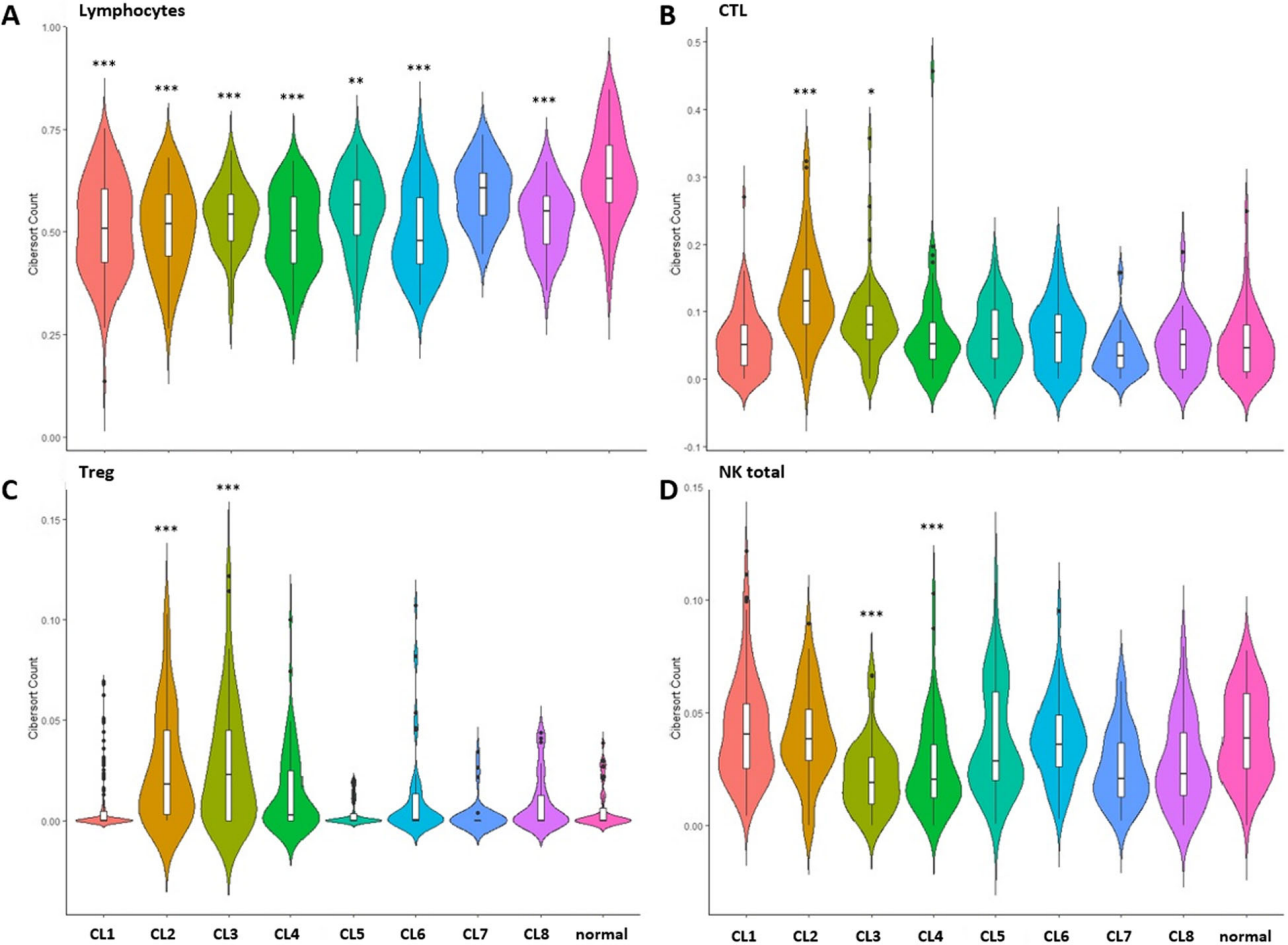
Immune cell abundance in the eight identified immune clusters. The distribution of lymphocyte abundance (A), cytotoxic T lymphocytes (CTL) (B), regulatory T cells (Treg) (C), and total natural killer (NK) cells (D). The asterisks indicate statistical significance compared to the normal tissues (* *p*-value < 0.05; ** *p*-value < 0.01; *** *p*-value < 0.001)

CIBERSORT was used to estimate the immune composition of the prostate samples used (Additional File 4). Using the CIBERSORT results for prostate samples, we calculated the total number of lymphocytes by totaling the abundance of the lymphocyte population (B cells, T cells, and NK cells) according to the method described by Thorsson et al. [24] (Fig. 1A and Additional file 4). The data was displayed using violin plots that were generated using the *ggplot2* package in R [25].

### Differential gene expression analysis

A differential gene expression analysis was performed using the *DESeq2* package in R (Additional File 3 and 5) [26]. Differentially expressed genes were those with an adjusted p-value less than 0.05 and a log_2_ fold change greater than 2. Genes with a log_2_ fold change less than 2 were considered to be minimally differentially expressed.

### Pathway analysis

To identify significantly enriched processes and pathways, we conducted an enrichment analysis on immune-related pathways from KEGG and GO terms in the R/Bioconductor packages *Pathview* and *Gage* [27, 28]. The pathway analysis was done in comparison to adjacent tissue samples (Additional files 6 and 7) and tumor tissue samples (Additional files 8 and 9).

### Fisher’s exact test

To identify whether a cluster is significantly associated with any clinical data, we performed Fisher’s exact test. The *p*-values were calculated by comparing the number of patients in a cluster belonging to a specific subtype to the total number of patients in the cluster. A *p*-value ≤0.05 indicates that the distribution of the number of patients in that cluster is significantly different from the overall pattern.

### Classification tree

To identify the biomarkers specific to each cluster, a classification tree was used to build a model to predict the immune evasion cluster into which a patient sample belongs. This was achieved using the *rpart* package in R [29].

## Results

### Patient cohort and gene expression data

The RNA-seq data for 498 prostate cancer samples, 51 non-malignant adjacent samples, and the associated de-identified patient information were obtained from TCGA. The RNA-seq data obtained from TCGA were reviewed for the expression of 2000 immune-related genes identified in previously published gene sets and the Molecular Signatures Database (MSigDB) (Additional File 2) [20–22]. The patients were then clustered sequentially using our sequential biclustering method to categorize the patients based on the expression of various immune genes [13]. The algorithm clustered 86.3% of the prostate cancer population into eight different immune clusters characterized by different combinations of immune evasion mechanisms (IEMs) (Tables 1 and 2, Additional file 10).

**Table 1:** The eight identified immune clusters in prostate cancer

	Genes	Number of Patients 430 (86.34%)
Cluster 1	232	129 (25.9%)
Cluster 2	314	44 (10.2%)
Cluster 3	276	52 (12.1%)
Cluster 4	129	56 (13.0%)
Cluster 5	116	43 (10.0%)
Cluster 6	27	49 (11.4%)
Cluster 7	111	28 (6.5%)
Cluster 8	52	29 (6.7%)

**Table 2:** The mechanisms of evasion in the identified prostate cancer clusters and the potential immunotherapies to circumvent immune evasion

Cluster	Mechanism of Evasion	Potential Immunotherapies
Cluster 1	Counterattack: DcR3 Ignorance	Anti-DcR3 Sipuleucel-T/DC-vaccines
Cluster 2 (Gleason 7)	Impaired antigen presentation/low activation of CTL Tolerance: CTLA4, PD-1 Counterattack: DcR3	Sipuleucel-T/DC-vaccines Anti-CTLA4, anti-PD-1 Anti-DcR3
Cluster 3 (Pathologic T3 & T4 Gleason score ≥ 8)	Tolerance: CTLA4, PD-1 Ignorance	Anti-CTLA4, anti-PD-1 Sipuleucel-T/DC-vaccines
Cluster 4 (Gleason score ≥ 8)	Tolerance: CTLA4 Ignorance	Anti-CTLA4 Sipuleucel-T/DC-vaccines
Cluster 5	Ignorance	Sipuleucel-T/DC-vaccines
Cluster 6	Tolerance: CTLA4 Counterattack: DcR3 Ignorance	Anti-CTLA4 Anti-DcR3 Sipuleucel-T/DC-vaccines
Cluster 7 (Gleason score 7)	Ignorance	Sipuleucel-T/DC-vaccines
Cluster 8	CTLA4 Ignorance	Anti-CTLA4 Sipuleucel-T/DC-vaccines

*DcR3* Decoy receptor 3, *DC* dendritic cell, *PD-1* programed cell death 1, *CTLA4* cytotoxic T lymphocyte associated protein 4

### Eight immune clusters based on different combinations of IEMs

**Fig. 2:**
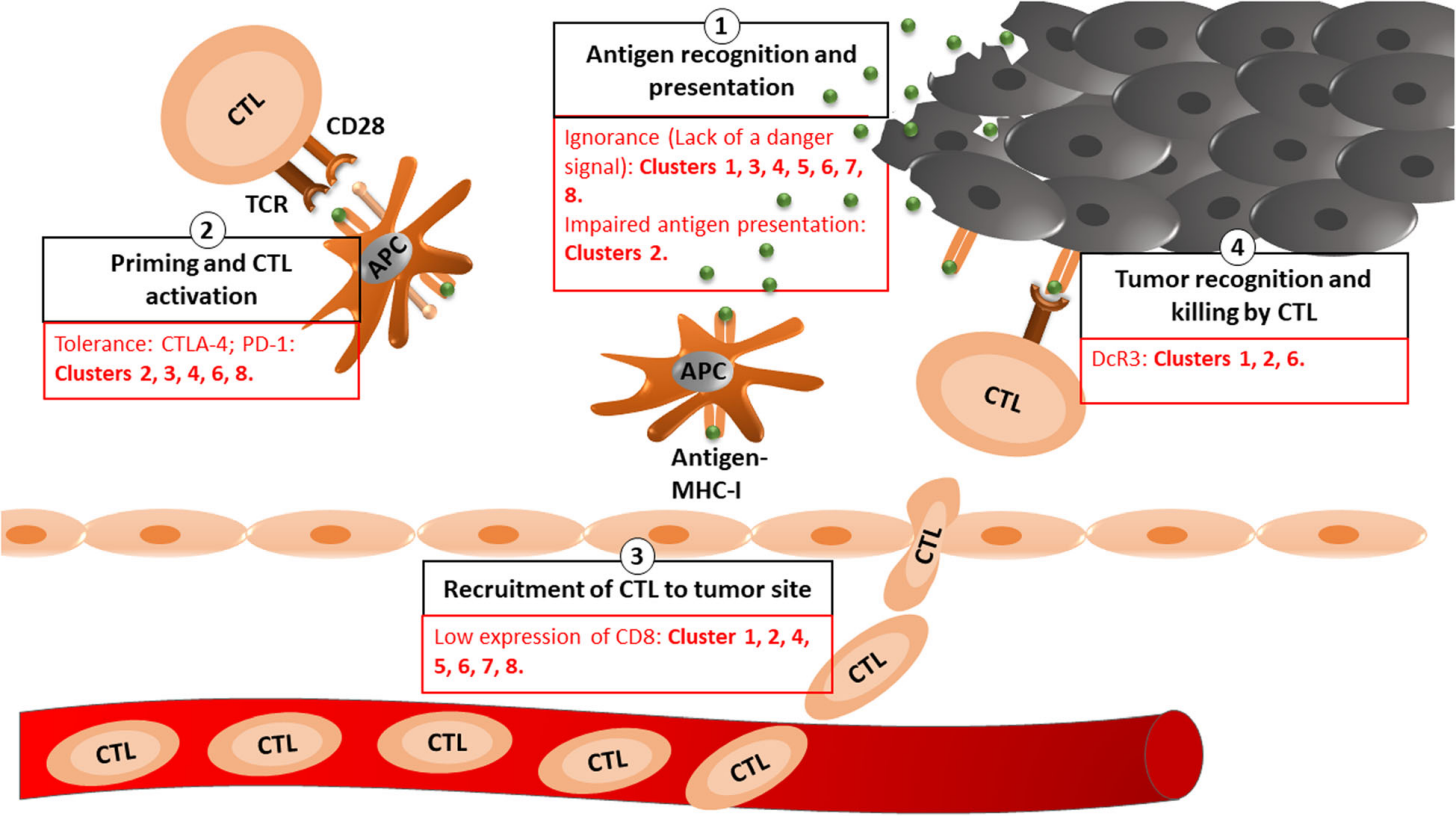
The different IEMs in prostate cancer at different steps of the cancer-immunity cycle. After analyzing the levels of gene expression of immune-related genes in the clusters compared to adjacent normal samples, we identified the IEMs activated at different steps of the cancer-immunity cycle

The gene expression data for each of the eight identified immune clusters were checked against the mean gene expression of non-malignant adjacent normal samples using the *DESeq2* package in R [26]. Differentially expressed genes were then analyzed using pathway analysis tools at the single gene level to identify any immune evasion mechanisms within the cancer-immunity cycle (Fig. 2 and Additional files 5, 6, 7, and 10).

The pathway analysis comparing clusters to the adjacent normal samples revealed that cluster 3 has a significant activation of the T helper 1 and T helper 2 differentiation pathway and T cell receptor (TCR) signaling. Additionally, cluster 5 showed significant downregulation in the interleukin-17 (IL-17) signaling pathway compared to the normal tissues (Additional files 6).

When investigating the immune cell abundance in each of the different clusters, we found that cluster 3 has the second highest cytotoxic lymphocyte (CTL) infiltration after cluster 2 (Fig. 1B, Additional files 4 and 11). Despite the higher activation of T helper cells, TCR signaling, and CTL infiltration in cluster 3 compared to other clusters, cluster 3 was significantly associated with a high Gleason score (≥ 8) and late pathologic-T stages (3 and 4) (Tables 3 and 4). This finding can be attributed to the highly immunosuppressive tumor microenvironment as demonstrated by the high infiltration of regulatory T cells (Treg) and upregulated expression of CTLA4 and PD-1 in this cluster (Fig. 1C, Table 2, and Additional files 3 and 10).

**Table 3:** Cluster association with the pathologic T stage

Cluster	Number of patients	T1	T2	T3	T4	Fisher exact p-value
Cluster 1	127	0 (0%)	51 (40.16%)	74 (58.27%)	2 (1.57%)	7.68E-01
Cluster 2	44	0 (0%)	23 (52.27%)	21 (47.73%)	0 (0%)	1.38E-01
Cluster 3	51	0 (0%)	9 (17.65%)	37 (72.55%)	5 (9.80%)	1.93E-03
Cluster 4	56	0 (0%)	16 (28.57%)	39 (69.64%)	1 (1.79%)	4.00E-01
Cluster 5	43	0 (0%)	19 (44.19%)	23 (53.49%)	1 (2.33%)	6.19E-01
Cluster 6	48	0 (0%)	16 (33.33%)	32 (66.67%)	0 (0%)	6.34E-01
Cluster 7	28	0 (0%)	15 (53.57%)	12 (42.86%)	1 (3.57%)	1.42E-01
Cluster 8	28	0 (0%)	8 (28.57%)	19 (67.86%)	1 (3.57%)	4.70E-01
Total	425	0 (0%)	183 (43.06%)	256 (60.24%)	12 (2.82%)

**Table 4:** Cluster association with the Gleason score

Cluster	Number of patients	Gleason score ≤ 6	Gleason score = 7	Gleason score ≥ 8	Fisher exact p-value
Cluster 1	129	11 (8.53%)	59 (45.74%)	59 (45.74%)	6.91E-01
Cluster 2	44	4 (9.09%)’	34 (77.27%)	6 (13.64%)	3.52E-04
Cluster 3	52	3 (5.77%)	17 (32.69%)	32 (61.54%)	2.75E-02
Cluster 4	56	1 (1.79%)	24 (42.86%)	31 (55.36%)	4.24E-02
Cluster 5	43	7 (16.28%)	19 (44.19%)	17 (39.53%)	3.38E-01
Cluster 6	49	9 (18.37%)	19 (38.78%)	21 (42.86%)	1.26E-01
Cluster 7	28	2 (7.15%)	24 (85.71%)	2 (7.14%)	2.13E-04
Cluster 8	29	4 (13.79%)	15 (51.72%)	10 (34.48%)	6.12E-01
Total	430	41 (9.54%)	211 (49.07%)	178 (41.39%)	

Although both clusters 5 and 7 and clusters 4 and 8 share the same immune evasion mechanisms (IEM), they exhibited differential gene expression (Additional files 3 and 10). Furthermore, cluster 4 was significantly associated with a Gleason score ≥ 8 whereas cluster 8 showed no significant association with a Gleason score. Similarly, cluster 7 had a Gleason score of 7 while cluster 5 did not (Table 4). In addition, the pathway analysis shows downregulated activation of IL-17 signaling in cluster 5 compared to the normal tissues and other prostate cancer patients. In contrast, cluster 4 showed downregulated activation of IL-17 signaling only when compared to other prostate cancer patients (not significantly lower than the normal tissues) (Additional files 7 and 9). Thus, further investigations regarding the role of IL-17 may shed light on its effect on the advancement of prostate cancer.

### Immunological ignorance, CTLA4, and DcR3 over-expression are the major evasion mechanisms in prostate cancer

Due to the low expression levels of the genes involved in antigen processing and presentation, immune cell recruitment, and immune activation, the majority of the clustered prostate cancer patients (89.77%) exhibited immunological ignorance [13, 17] (Table 2, Additional files 5 and 10). This finding is in line with the identification of prostate cancer as a poorly immunogenic disease. Ignorance can result from either the absence of tumor-specific antigens that activate the immune system or the failure of APCs to recognize cancer antigens.

CTLA4-mediated immune tolerance and a counterattack with DcR3 were identified in 58.8 and 51.6% of the clustered patients, respectively. Upregulated PD-1 expression (27.8%) was accompanied by upregulated CTLA4 expression in prostate cancer patients (Fig. 1C, Additional file 10). Interestingly, 41.4% of patients with immunologic ignorance also showed upregulated DcR3 expression, 43.26% displayed upregulated CTLA4 expression, and 11.4% exhibited upregulated expression of all three molecules. Ignorance and upregulated PD-1 expression were identified in 12.09% of the clustered patients. Thus, some patients could respond to cellular immunotherapy alone, while others may require combined or sequential therapy with anti-CTLA4, anti-DcR3, or anti-PD-1. However, these results need to be further validated clinically.

### Late-stage disease and high Gleason score in cluster 3

Despite the higher level of cytotoxic T-lymphocyte infiltration observed in clusters 2 and 3, the tumors had high levels of PD-1, CTLA4, and Tregs (Table 2, Fig. 1B and Fig. 1C), indicating an immunosuppressive status. Moreover, advanced prostate cancer was significantly associated with clusters 3 and 4, whose mean NK cell counts were significantly downregulated compared to normal (Fig. 1D and Additional file 8). Both clusters showed a significant association with a Gleason score ≥ 8. Cluster 3 was also significantly associated with pathologic-T stages 3 and 4 and a higher Treg cell abundance than cluster 4 (Tables 3 and 4). Evasion mechanisms identified in these clusters consisted of a combination of tolerance and impaired antigen presentation or ignorance (Table 2).

### Identification of 10 predictive biomarkers for patient stratification

**Fig. 3:**
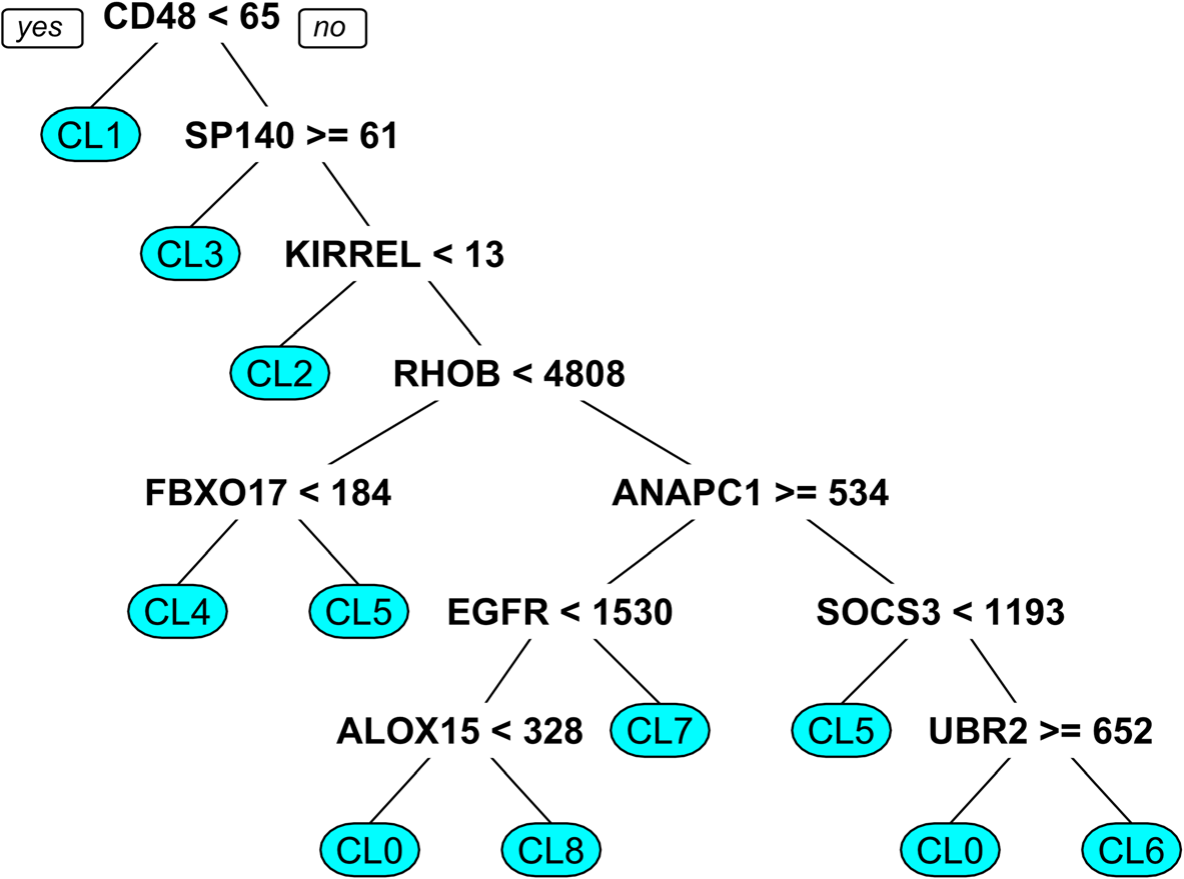
Classification tree with 10 predictive biomarkers of patients’ immune evasion clusters (CL) and response to immunotherapy. Cluster of differentiation 48 (CD48), Speckled 140 KDa (SP140), Kin Of IRRE Like (KIRREL), Rho-Related GTP-Binding Protein RhoB (RHOB), F-Box Protein 17 (FBXO17), Anaphase Promoting Complex Subunit 1 (ANAPC1), Epidermal growth factor receptor (EGFR), Suppressor Of Cytokine Signaling 3 (SOCS3), Arachidonate 15-Lipoxygenase (ALOX15), Ubiquitin Protein Ligase E3 Component N-Recognin 2 (UBR2)

After the identification of eight different immune evasion clusters, we next sought to select biomarkers that could predict which patient populations would be most likely to respond to various immunotherapies. A classification tree model was built to predict a patient’s membership to a specific immune evasion cluster. The classification tree achieved an accuracy of 77%. The selected gene biomarkers and their expression cutoff values are displayed in Fig. 3. These biomarkers are CD48, SP140, KIRREL, RHOB, FBXO17, ANAPC1, EGFR, SOCS3, ALOX15, and UBR2. Cluster 1 is distinguished from all other clusters, especially similar clusters 2 and 3 (close nodes in the tree), by its CD48 expression, which is less than 65 reads. CD48 is a member of the signaling lymphoid activation molecule family (SLAM) which is important for adhesion and activation of immune cells and plays a role in tolerance and immunity [30]. This explains the absence of tolerance IEM in cluster 1 compared to other neighboring node clusters in the tree, such as clusters 2 and 3, due to its lower CD48 expression. Cluster 3 is identified by its higher CD48 expression compared to cluster 1 and increased expression of lymphoid-specific SP100 homolog (SP140), which is a repressor of inflammation, cell-cell adhesion, and nuclear factor kappa-light-chain-enhancer of activated B cells (NF-κB) regulated pathways [31]. Furthermore, to distinguish cluster 3 from all other clusters, and thus identify the correct combination immunotherapy for these patients, 3 biomarkers are required: CD48, SP140, and KIRREL (Table 2 and Fig. 3). Another important finding from the identified biomarkers is that even for the clusters that have upregulated CTLA4 and PD-1 expressions, these molecules are not the optimal biomarkers for the choice of anti-CTLA4 or anti-PD-1 treatments. Additional file 12 further addresses the roles of each of our identified biomarkers.

## Discussion

Prostate cancer immunotherapy is an underexplored area of research due to the misconception that prostate cancer is non-immunogenic. A paradigm shift began in the 1990s when preclinical and subsequent clinical and translational research showed that some prostate cancers respond to immune modulators [32]. Clinical trials and other studies starting in the 1990s have found that high dose IL2, IFN-α, and IFN-ϒ induced objective PSA response in metastatic prostate cancer. Furthermore, GVAX cancer vaccine combination therapy with anti-CTLA4, GM-CSF-activated dendritic cell-based antigen presentation, and other vaccinia-based treatments showed improved survival and immunogenicity [32–34]. While none of these treatments have made it to FDA approval due to their unsatisfactory results, they paved the way for therapeutic approaches that are aimed at both increasing tumor recognition by immune cells to elicit an anti-tumor immune response, as well as counter-acting immunosuppression. Sipuleucel-T and Keytruda are the immunotherapies approved by the FDA for metastatic prostate cancer. While Sipuleucel-T administration improved overall survival, it did not show a difference in the progression-free survival in the treatment group compared to placebo [6], urging the need for more immunotherapy clinical trials to improve the current outcomes. Keytruda however was approved for solid tumors with MMR mutations and/or MSI and was only given for prostate cancer patients once they have shown no response to any other available treatment. While several trials are currently ongoing (Additional file 1) the gap lies in the lack of available biomarkers that can help predict which patients would best respond to a particular immunotherapy or combination of therapies.

To close this gap, we clustered prostate cancer patients into eight groups based on their patterns of immune gene expression and identified the associated IEMs and biomarkers that are predictive of a patient’s IEM cluster (Figs. 2 and 3). Since the current approach for giving anti-CTLA4 and anti-PD-1 treatments that are based on the patient’s level of expression of CTLA4 and PD-1 have failed, the need for better biomarkers is necessary for improving immunotherapy outcomes. Our identified biomarkers, which did not include either CTLA4 or PD-1, may further corroborate that these molecules are not ideal biomarkers for treatment selection. Thus, our approach not only facilitates a more personalized approach to immunotherapy based on a patient’s IEM but also provides possible reasons behind the failure of several mono-immunotherapeutic approaches in prostate cancer. Prostate cancer patients with upregulated CTLA4 or PD-1 expression also exhibited other immune evasion mechanisms that obstruct the cancer immunity cycle. The clusters that showed upregulated PD-1 expression (34.3%) also exhibited upregulated CTLA4 (34.3%). Such expression was associated with either immunologic ignorance (cluster 3, 12.1% of clustered patients) or impaired antigen presentation and upregulated DcR3 (cluster 2, 10.23% of clustered patients). Similarly, the clusters with upregulated CTLA4 all possessed immunologic ignorance as an additional evasion mechanism. Thus, as clinical trials have shown, targeting CTLA4 or PD-1 alone will not succeed in treating prostate cancer [6, 8, 9, 35]. A combined immunotherapy approach based on a patient’s immune evasion cluster may be more likely to result in a favorable response rate.

Immunotherapy is typically avoided during early-stage prostate cancer given the relatively successful hormonal and surgical options; however, castrate-resistance and hormone-refractory disease may be avoided if hormone deprivation therapy is administered in combination or sequentially with an immune system booster to delay disease progression. Here, we showed that untreated prostate cancer tissue samples have various combinations of evasion mechanisms, which if targeted early, may result in better efficacy compared to the conventional treatment options alone. The neo-adjuvant administration of Sipuleucel-T before a prostatectomy was shown to elicit a systemic antigen-specific immune response and increase T cell infiltration into the tumor microenvironment [36]. The Checkmate 650 trial also showed that the cohort treated with a combination of anti-PD-1 and anti-CTLA4 before chemotherapy showed greater benefit than the group that received chemotherapy alone [12].

Sipuleucel-T is more likely to benefit patients with immunologic ignorance, which was identified in the majority of the clustered patients (89.77%). However, immunologic ignorance was associated with other evasion mechanisms in all clusters, except clusters 5 and 7 (16.5%). The administration of Sipuleucel-T alone to patients in clusters 5 and 7 may substantially improve the response rate. Patients in other clusters can be treated with Sipuleucel-T in combination with immune checkpoint inhibitors. However, the active evasion mechanisms in a cancer patient must be monitored regularly after treatment to identify any newly developed evasion mechanisms and target them with the relevant immunotherapies.

Although certain clusters were found to share the same evasion mechanisms, the extent of these mechanisms varies based on the differential expression of the associated genes. This was clearly shown in the classification tree that distinguished clusters 4 and 8 and clusters 5 and 7 with different sets of biomarkers (Fig. 3). Furthermore, the Gleason scores were different in both clusters 5 and 7 and clusters 4 and 8, and the clusters exhibited a different activation/deactivation status of the IL-17 signaling pathway. The role of IL-17 in carcinogenesis has long been controversial and IL-17 has been proposed to have pro-tumor and anti-tumor roles by increasing the tumor vasculature and aiding in metastasis, as well as increasing the infiltration of immune cells, respectively [37–41]. However, IL-17 was found to promote prostate cancer in mice and human cell lines by inducing the epithelial to mesenchymal transition via matrix metalloproteinase-7 (MMP-7) [42–44]. IL-17 enhancement of prostate adenocarcinoma in castration-resistant prostate cancer in a mouse model was attributed to potential creation of immunotolerant and pro-angiogenic tumor microenvironment [43]. Furthermore, IL-17 was found to recruit myeloid-derived suppressor cells (MDSCs) and increase the immunosuppressive effects of MDSCs on T cells, creating an immunotolerant tumor microenvironment [43–46]. Thus, further investigation into the effect of IL-17 on immune evasion can help elucidate whether it could be a potential immunotherapeutic target or a prognostic biomarker.

## Conclusions

The stratification of prostate cancer patients using the biomarkers discovered in this study allows for a more precise grouping of patients for monotherapy or combination therapy testing. Although these proposed therapeutic approaches need to be further validated clinically, we believe that this personalized approach may improve the currently disappointing immunotherapy outcomes in prostate cancer. While combination therapy may result in poor tolerability, further studies regarding the combination regimen, specifically the administration and dose sequence, may alleviate their side effects. Thus, improving the success of immunotherapy in prostate cancer may be possible by both ensuring that the cancer-immunity cycle remains activated and by targeting immunosuppressive molecules that prevent its self-amplification using personalized strategies.

## Supplementary information

**Additional file 1.** Prostate cancer immunotherapy clinical trials. This file contains combination and mono-immunotherapies in clinical trial.

**Additional file 2.** List of immune genes. The file contains a list of 2000 immune gene collected from MSigDB and from other publications.

**Additional file 3.** The identified immune evasion clusters. This file shows for every cluster the list of patients and their corresponding levels of gene expression for all the investigated immune genes.

**Additional file 4.** Immune cell abundance for each patient in the 8 clusters. The abundance of immune cells was identified using CIBESORT for the patients in the identified clusters.

**Additional file 5.** Differential gene expression of the clusters compared to normal adjacent tissues. Differential gene expression analysis using DESeq are shown along with p-values and log2 fold change.

**Additional file 6.** Pathway analysis using gene ontology (GO) for the tumor-normal comparison.

**Additional file 7.** Pathway analysis using KEGG pathway for the tumor-normal comparison.

**Additional file 8.** Pathway analysis using GO for the tumor-tumor comparison.

**Additional file 9.** Pathway analysis using KEGG pathway for the tumor-tumor comparison.

**Additional file 10.** Differential gene expression for the analysis of the cancer-immunity pathway. The genes important for each step of the cancer immunity pathway were organized for the identification of the IEMs in each cluster.

**Additional file 11.** Statistical analysis of immune cells. The differential abundance of cells between the clusters and normal tissue samples was done using pairwise T-test in R.

**Additional file 12.** The identified biomarkers and their functions. The identified biomarkers from the decision tree and their functions are shown in this file

## Data Availability

All the data was taken from the cancer genome Atlas for prostate adenocarcinoma samples (PRAD). All generated clusters and analyses from this study are included in the published article and its supplements.

## Abbreviations

ALOX15: Srachidonate 15-lipoxygenase
ANAPC1: Anaphase promoting complex subunit 1
APC: Antigen-presenting cell
CD48: Cluster of differentiation 48
CEA: Carcinoembryonic antigen
Cl: Cluster
CTL: Cytotoxic T lymphocyte
CTLA4: Cytotoxic T-lymphocyte-associated protein 4
DcR3: Decoy receptor 3
EGFR: Epidermal growth factor receptor
FBXO17: F-box protein 17
GM-CSF: Granulocyte-macrophage colony-stimulating factor
IDO: Indoleamine 2,3-dioxygenase 1
IEM: Immune evasion mechanism
IEMA: Immune evasion mechanism analysis
IL: Interleukin
IFN: interferon
KIRREL: Kin of IRRE like
mCRPC: Metastatic castrate-resistant prostate cancer
MDSC: Myeloid-derived suppressor cells
MMP-7: Matrix metalloproteinase-7
MMR: Mismatch repair genes
MSigDB: Molecular Signatures Database
MSI: Microsatellite instability
MUC1: Mucin 1
NF-κB: Nuclear factor kappa-light-chain-enhancer of activated B cells
NK cell: Natural killer cell
PD-1: Programmed Cell Death 1
PD-L1/2: Programmed death-ligand 1/2
PRAD: Prostate adenocarcinoma
PSA: Prostate-specific antigen
RHOB: Rho-related binding protein RhoB
SLAM: Signaling lymphoid activation molecule family
SOCS3: Suppressor of cytokine signaling 3
SP140: Speckled 140KDa
TCGA: The Cancer Genome Atlas
TCR: T cell receptor
TGF-β: Transforming growth factor-beta
TRAILR4: TNF-Related Apoptosis-Inducing Ligand Receptor 4
Treg: Regulatory T cell
UBR2: Ubiquitin protein ligase component N-recognin 2
